# SMAP29: an antibacterial peptide that possesses anti-inflammatory and fast bactericidal actions against colistin-resistant gram-negative bacteria

**DOI:** 10.1128/spectrum.02808-25

**Published:** 2026-05-05

**Authors:** Ziyue Zeng, Mengjie Wei, Deyi Zhao, Huijing Zhou, Endian Sun, Xiaowei Lv, Yuhan Yang, Tieli Zhou, Chunquan Xu

**Affiliations:** 1 Department of Clinical Laboratory, The First Affiliated Hospital of Wenzhou Medical University; Key Laboratory of Clinical Laboratory Diagnosis and Translational Research of Zhejiang Province 89657, Wenzhou, Zhejiang, China; 2 School of Laboratory Medicine and Life Science, Wenzhou Medical University 26453, Wenzhou, China; Emory University School of Medicine, Atlanta, Georgia, USA

**Keywords:** colistin, antimicrobial peptide, SMAP29, multidrug-resistant, gram-negative bacteria

## Abstract

Multidrug-resistant (MDR) gram-negative bacteria (GNB) are threatening global public health. Since colistin (COL) is the last resort antibiotic for MDR gram-negative infections, the rise in colistin-resistant ‌(COL-R) bacteria could pose risks to people. The current research investigation explored the antimicrobial, anti-biofilm, and anti-inflammatory capacities of SMAP29, a naturally generated cationic antibacterial peptide, against clinical COL-R *Klebsiella pneumoniae*, *Pseudomonas aeruginosa*, *Escherichia coli*, *Acinetobacter baumannii*, and explored its antibacterial mechanisms. The results demonstrated that SMAP29 exhibited a very low minimum inhibitory concentration against COL-R GNB and rapidly killed bacteria within 30 min. In addition, SMAP29 inhibited biofilm growth and eliminated it. According to another study’s findings, SMAP29 could lead to the membrane’s integrity being wiped out, which could result in the formation of intracellular reactive oxygen species. Moreover, SMAP29 could effectively prevent RAW 264.7 macrophages in mice from generating the pro-inflammatory cytokines TNF-α, IL-6, and IL-1β. Assays for cytotoxicity and hemolysis *in vitro* revealed that SMAP29 proved safe at the tested concentrations. *In vivo* experiments further demonstrated that the amount of bacteria in the infected mice’s thighs was significantly reduced through SMAP29 treatment. Collectively, our findings demonstrate that SMAP-29’s efficacy stems from its dual action: rapid bactericidal activity via lipopolysaccharide-mediated membrane disruption and concurrent anti-inflammatory effects. This multifaceted potency, validated both *in vitro* and *in vivo*, positions SMAP-29 as a promising therapeutic candidate against multidrug-resistant bacterial infections.

**IMPORTANCE:**

Multidrug-resistant gram-negative bacteria have emerged as critical threats to global public health, driving intractable infections with steadily rising incidence. These pathogens exhibit formidable tolerance to virtually all classes of contemporary antibiotics, translating into persistently high mortality and formidable clinical challenges. This study elucidates the antibacterial, anti-biofilm, and anti-inflammatory activities of the antimicrobial peptide SMAP-29 against colistin-resistant gram-negative organisms while dissecting its underlying mechanisms. Our findings provide a mechanistic framework and a promising therapeutic avenue for combating multidrug-resistant infections.

## INTRODUCTION

Multidrug-resistant (MDR) microorganisms have evolved into a global health threat in recent years. Colistin (COL) is the final option for gram-negative bacterial infections (1, 2). However, colistin-resistant (COL-R) gram-negative bacteria (GNB) have become prevalent around the world in parallel with the clinical application of COL. COL resistance is becoming a bigger issue globally, especially in Asia and the Middle East (3, 4). World Health Organization (WHO) continues to focus on this issue through its Global Antimicrobial Resistance Monitoring System and other reports and highlights the pressing necessity of creating fresh antibacterial strategies to deal with this issue (5).

Antimicrobial peptides (AMPs) constitute an integral component of the innate immune system across numerous animal species, serving as a critical defense mechanism against microbial infections (6, 7). They are cationic and amphiphilic compounds with fewer than 50 amino acid residues (8, 9). AMPs have the capability to kill many species of microorganisms, encompassing bacteria, fungi, and viruses, among others (10, 11). Numerous AMPs also exhibit a certain effect on MDR bacteria (12, 13). Lipopolysaccharide (LPS) is a unique component of the outer membrane of gram-negative bacteria, composed of lipid A, core oligosaccharide, and O-antigen. Some antimicrobial peptides (such as PvML1) can neutralize LPS and regulate inflammation-related signaling pathways (14). In addition, AMPs demonstrate excellent thermal stability, absence of teratogenic effects, negligible toxicity accumulation, and low propensity for inducing drug-resistant strains (15). Therefore, AMPs, with their aforementioned advantages, hold promise as a new generation of antibacterial agents.

SMAP29 is an α-helix antimicrobial peptide derived from mRNA of sheep bone marrow cells, which is produced by sheep myeloid cells (16). The structure of SMAP29 comprises an α-helix at the N end, a hinge region of Gly-Pro, and a hydrophobic segment at the C end (17, 18). Nevertheless, the precise target and mechanism of its antibacterial activity, how SMAP29 inhibits macrophages’ production of inflammatory cytokines caused by LPS is unknown (19).

In diverse GNB strains with varying levels of COL resistance, the study compares the antibacterial and anti-inflammatory properties of SMAP29 and COL. In addition, *in vivo* studies were conducted to assess SMAP29’s antibacterial capacity in a mouse infection model.

## RESULTS

### Rapid bactericidal effect of antimicrobial peptide SMAP29 on colistin-resistant gram-negative bacteria

In Table 1, 32 clinically COL-R GNB were randomly screened. SMAP29 has a minimum inhibitory concentration (MIC) in the spectrum of 0.25–4 μg/mL against COL-R GNB, indicating that the MIC of SMAP29 is lower than that of colistin at the same concentration.

**TABLE 1:** Common clinical antibiotics and SMAP29 MICs against COL-R GNB

Species	Strains^*a*^	Antibiotics^*b*^								SMAP29
		Breakpoints (S-R)^*c*^ MIC (mg/L)								
		ATM	FEP	IPM	CIP	LVX	GEN	TOB	COL	
		4–16	2–16	1–4	0.25–1	0.5–2	4–16	4–16	2–4	
*E. coli*	DC90	**≥256**	**64**	**≥256**	**64**	**32**	**≥256**	**128**	**4**	2
	DC19840	**4**	4	≤1	≤0.25	≤0.25	≤1	≤1	**8**	0.5
	DC18824	**4**	**2**	≤1	**≥4**	**≥8**	≤1	≤1	**32**	0.25
	DC3846	**128**	**≥256**	0.5	**≥256**	**128**	**≥256**	**64**	**4**	0.5
	DC5286	**≥256**	**≥256**	0.25	**128**	**64**	4	4	**4**	0.25
	DC19144	≤1	≤1	≤1	**≥4**	**≥8**	≤1	≤1	**8**	2
	DC19526	≤1	≤1	≤1	**≥4**	**≥8**	≤1	≤1	**4**	2
	DC19829	≤1	≤1	≤1	**≥4**	**≥8**	**≥16**	8	**8**	1
*K. pneumoniae*	FK1913	**≥64**	**8**	≤1	**≥4**	**≥8**	**≥16**	**≥16**	**128**	2
	FK3994	**≥256**	**≥256**	**32**	**≥256**	**64**	**≥256**	**≥256**	**64**	2
	FK6556	**64**	**64**	**16**	**4**	**8**	**16**	**16**	**8**	0.5
	FK6663	**≥256**	**≥256**	**32**	**≥256**	**≥256**	**≥256**	**≥256**	**4**	1
	FK6696	**≥256**	**≥256**	**128**	**≥256**	**64**	**≥256**	**≥256**	**128**	4
	FK11237	≤1	≤1	**≥16**	≤0.25	≤0.25	≤1	≤1	**4**	1
	FK12771	**≥64**	**≥64**	**≥16**	**≥4**	**≥8**	**≥16**	**≥16**	**64**	2
	FK12716	≤1	≤1	≤1	≤0.25	≤0.25	≤1	≤1	**16**	0.5
		**-**	**8–32**	**2–8**	**1–4**	**2–8**	**4–16**	**4–16**	**2–4**	
	BM1342	64	**≥256**	≤0.25	**64**	**32**	1	1	**4**	0.5
*A. baumannii*	BM1412	128	**≥256**	**8**	**256**	**32**	8	1	**4**	1
	BM2431	64	**64**	**16**	**4**	**8**	1	1	**4**	1
	BM7477	64	**≥64**	**≥16**	**≥4**	4	**≥16**	**≥256**	**128**	0.5
	BM2349	≥8	**≥8**	**≥8**	**≥0.5**	**≥1**	**≥4**	**≥4**	**4**	1
	BM7994	32	**≥64**	**≥16**	**≥4**	4	**≥16**	**≥16**	**16**	1
	BM8014	16	≥8	**16**	**128**	**≥16**	**≥256**	**≥256**	**16**	1
	BM2370	16	**2**	≤1	≤0.25	≤0.25	≤1	≤1	**8**	1
		**8–32**	**8–32**	**2–8**	**0.5–2**	**1–4**	**4–16**	**4–16**	**2–4**	
	TL2314	**16**	8	≤1	0.5	2	8	≤1	**2**	1
*P. aeruginosa*	TL7333	**16**	**64**	2	2	**≥8**	**≥16**	**64**	**16**	4
	TL7440	**≥16**	**64**	**≥8**	**≥4**	**≥4**	**≥16**	0.5	**16**	2
	TL7505	4	≤0.25	≤1	**≥4**	2	2	0.5	**8**	4
	TL7548	4	**32**	2	0.5	0.5	2	≤1	**16**	1
	TL2917	**≥64**	**8**	**8**	0.5	1	≤1	≤1	**4**	1
	TL7929	4	**256**	4	**≥8**	≤0.25	2	1	**32**	4
	TL8126	4	**256**	**≥16**	0.5	0.5	≤1	≤1	**32**	4

^*a*^ Strains in boldface are MDR strains.

^*b*^ ATM, aztreonam; FEP, cefepime; IPM, imipenem; CIP, ciprofloxacin; LVX, levofloxacin; GEN, gentamicin; TOB, tobramycin; COL, colistin.

^*c*^ S-R represents the susceptible (S) breakpoint to resistant (R) breakpoint, according to CLSI supplement M100 (30th edition) and EUCAST.

**Fig 1:**
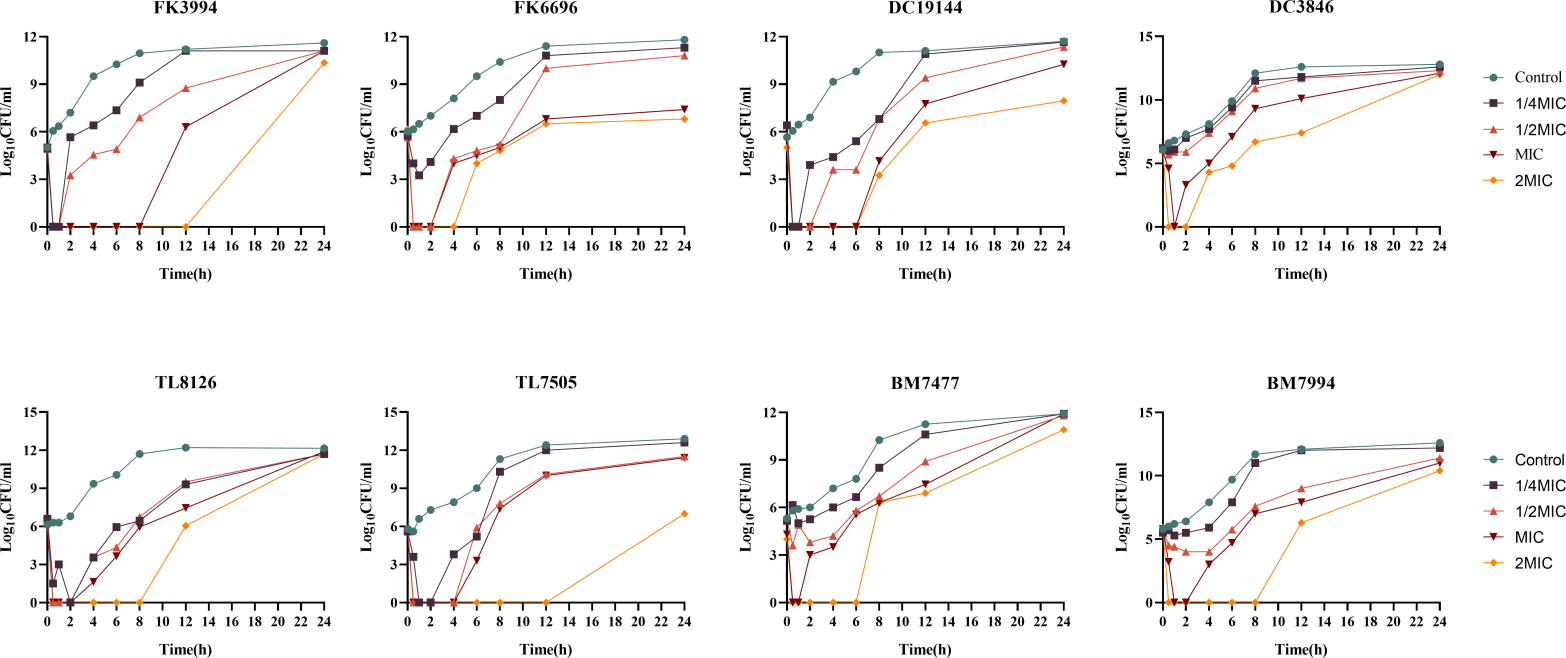
Time-kill kinetics of SMAP29 against gram-negative bacteria (*n* = 3). SMAP29 was tested against *Klebsiella pneumoniae* (FK), *Escherichia coli* (DC), *Pseudomonas aeruginosa* (TL), and *Acinetobacter baumannii* (BM) at concentrations of 1/4×, 1/2×, 1×, and 2× MIC.

Eight representative COL-R strains were selected for time-kill assays based on the MIC results from Table 1. As shown in Fig. S1, SMAP29 at 1× MIC achieved a >3 log₁₀ CFU/mL reduction within the first 10 min of exposure, demonstrating its exceptionally rapid bactericidal action. At the MIC level, SMAP29 maintained bacteriostatic activity for up to 12 h for most strains, while at 2× MIC, it exhibited sustained bactericidal efficacy over 24 h for several isolates (Fig. 1).

### Effect of antimicrobial peptide SMAP29 on bacterial biofilm

**Fig 2:**
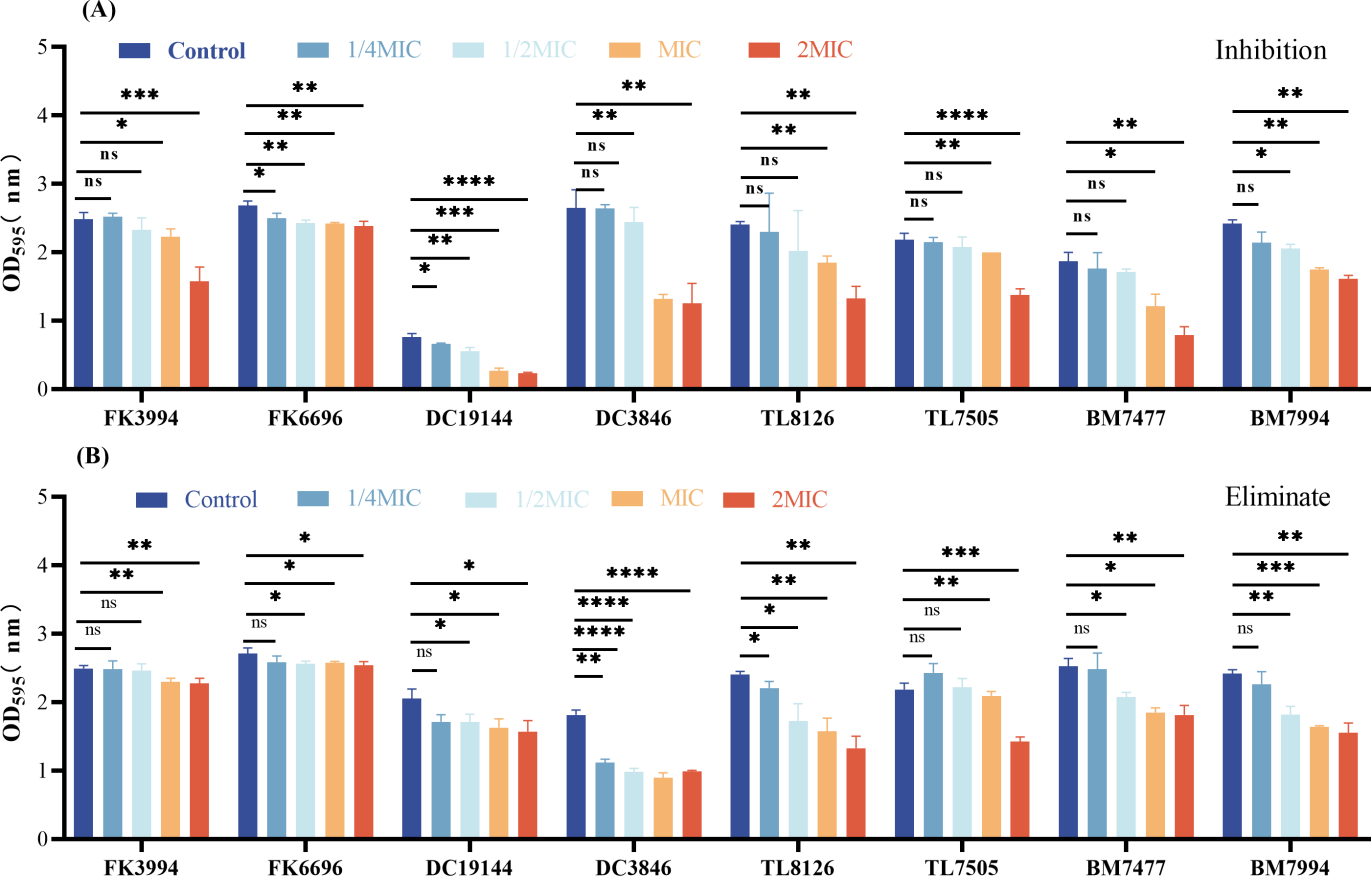
Effects of SMAP29 on biofilms of gram-negative bacteria (*n* = 3). (**A**) Inhibition of biofilm formation. (**B**) Removal of pre-formed biofilms. SMAP29 was tested at sub-inhibitory to inhibitory concentrations (1/4×, 1/2×, 1×, and 2× MIC) against *Klebsiella pneumoniae* (FK), *Escherichia coli* (DC)*, Pseudomonas aeruginosa* (TL), and *Acinetobacter baumannii* (BM). *P* < 0.05 (*), *P* < 0.01 (**), *P* < 0.001 (***), and *P* < 0.0001 (****); ns, not significant.

**Fig 3:**
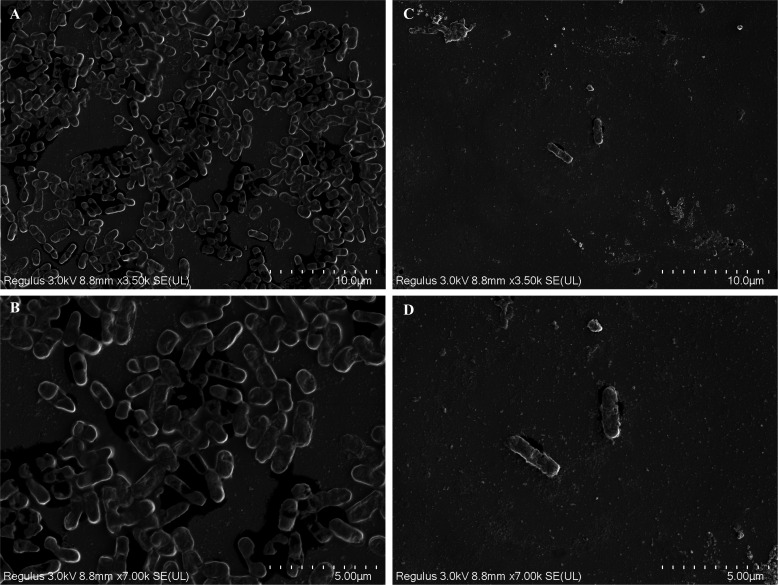
*E. coli* DC19144 SEM picture shows differences in biofilm and bacterial shape between groups. (**A**) MHB-control, 3,500×; (**B**) 7,000×; (**C**) SMAP29 (2 μg/mL), 3,500×; and (**D**) 7,000×.

Crystal violet staining was used to look at how SMAP29 impacted the growth and clearing of biofilms. In contrast to the control group, SMAP29 dramatically reduced the amount of biofilms in all experimental strains at MIC and 2× MIC (*P* < 0.05) (Fig. 2A). In Fig. 2B, SMAP29 was effective at both MIC and 2× MIC levels for disrupting established biofilm (*P* < 0.05), and the biofilm yields of the eight strains decreased in a way that was dependent on dosage. We further observed the inhibitory effect of SMAP29 on biofilm under the scanning electron microscope (SEM). The density and morphology of DC19144 biofilm treated with SMAP29 were observed by SME (Fig. 3). On the contrary, under the magnification of 3,500 times and 7,000 times, SMAP29 treatment with MIC concentration significantly reduced and destroyed bacterial biofilm and decreased the number of bacteria.

### SMAP29 promotes reactive oxygen species to be produced and affects the membrane’s integrity

**Fig 4:**
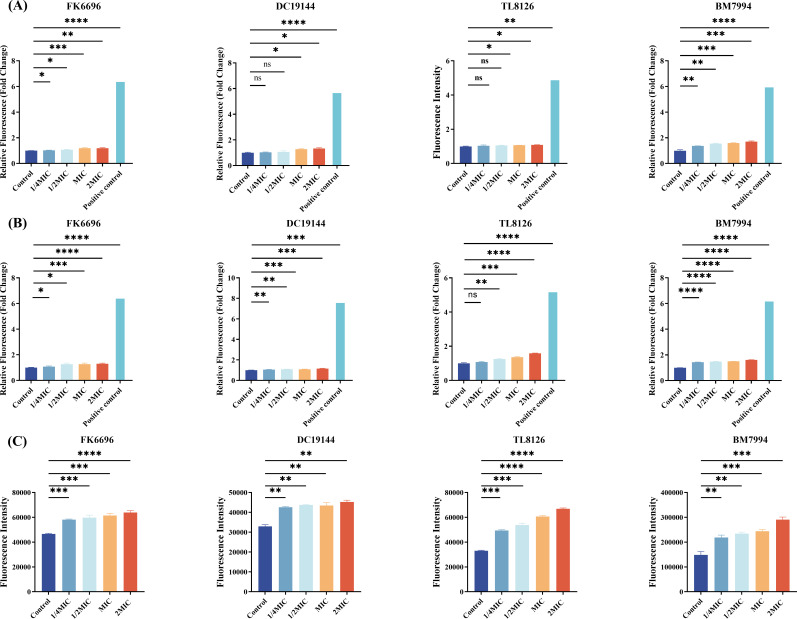
SMAP29 induces concentration-dependent membrane disruption and reactive oxygen species (ROS) generation in gram-negative bacteria. (**A**) Outer membrane (OM) permeability assessed by NPN uptake. (**B**) Inner membrane (IM) permeability assessed by propidium iodide (PI) uptake. (**C**) Intracellular ROS levels were detected using the fluorescent probe DCFH-DA. All strains*—Klebsiella pneumoniae* (FK), *Escherichia coli* (DC), *Pseudomonas aeruginosa* (TL), and *Acinetobacter baumannii* (BM)—were treated with SMAP29 at 1/4×, 1/2×, 1×, and 2× MIC. 0.1% Triton X-100 (**A and B**) served as positive controls. Fluorescence data are normalized to the respective untreated control (set as 1) and presented as mean ± SD of three independent experiments (*n* = 3). *P* < 0.05 (*), *P* < 0.01 (**), *P* < 0.001 (***), and *P* < 0.0001 (****); ns, not significant.

We carried out a variety of experiments using identical bacterial strains (FK6696, DC19144, TL8126, and BM7994) to determine SMAP29’s probable antibacterial mechanism. The first experiment concentrated on differences in membrane permeability. N-Phenyl-1-naphthylamine (NPN), a hydrophobic fluorescent dye, was used to test SMAP29’s role in bacterial outer membrane permeability in different doses. As shown in Fig. 4A, in contrast with controls, SMAP29 substantially or in a dose-dependent manner increased OM permeability.

The cells were also treated with PI dye to assess SMAP29’s effects on bacterial inner permeability. The PI’s ability to penetrate intact cell membranes is limited because only when the cell membrane is compromised can it enter the cytoplasm. When PI enters, it binds to DNA and then fluoresces. As the amount of SMAP29 increased, it also increased the fluorescence level of PI emission, showing that SMAP29 has the permeability to boost IM’s permeability (Fig. 4B).

Subsequent research was done to find out whether SMAP29 could harm bacteria in ways other than by rupturing the integrity of their cell membrane. Intracellular esterases have the ability to hydrolyze the 2′, 7′-dichlorodihydrofluorescein diacetate (DCFH-DA) fluorescent probe, which forms DCFH after being quenched in an extracellular environment. DCFH is oxidized by ROS to produce green fluorescent biological DCFH. Compared to the control group, the SMAP29 treatment group exhibited significantly higher intracellular ROS levels (Fig. 4C).

### SMAP29 can cause bacterial death

**Fig 5:**
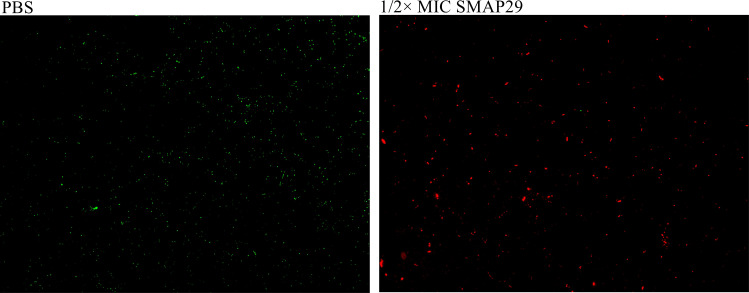
CLSM live/dead staining was performed to show the ability of SMAP29 to resist bacteria.

We used the confocal laser scanning microscopy (CLSM) live/dead staining technique for fluorescence dual staining of biofilms, which allowed the direct visualization of the antibiofilm capacity of SMAP29 therapy. To avoid inhibiting bacterial growth and biofilm formation, we conducted the experiment using a drug concentration of 1/2× MIC value and DC 19144 as the experimental isolate. The two-dimensional plan of bacteria revealed that the red fluorescence increased greatly under the action of sub-inhibitory concentration (Fig. 5), implying that the SMAP29 of sub-inhibitory concentration may result in the death of some bacteria in the biofilm.

### SMAP29 showed no toxicity at the tested concentrations

**Fig 6:**
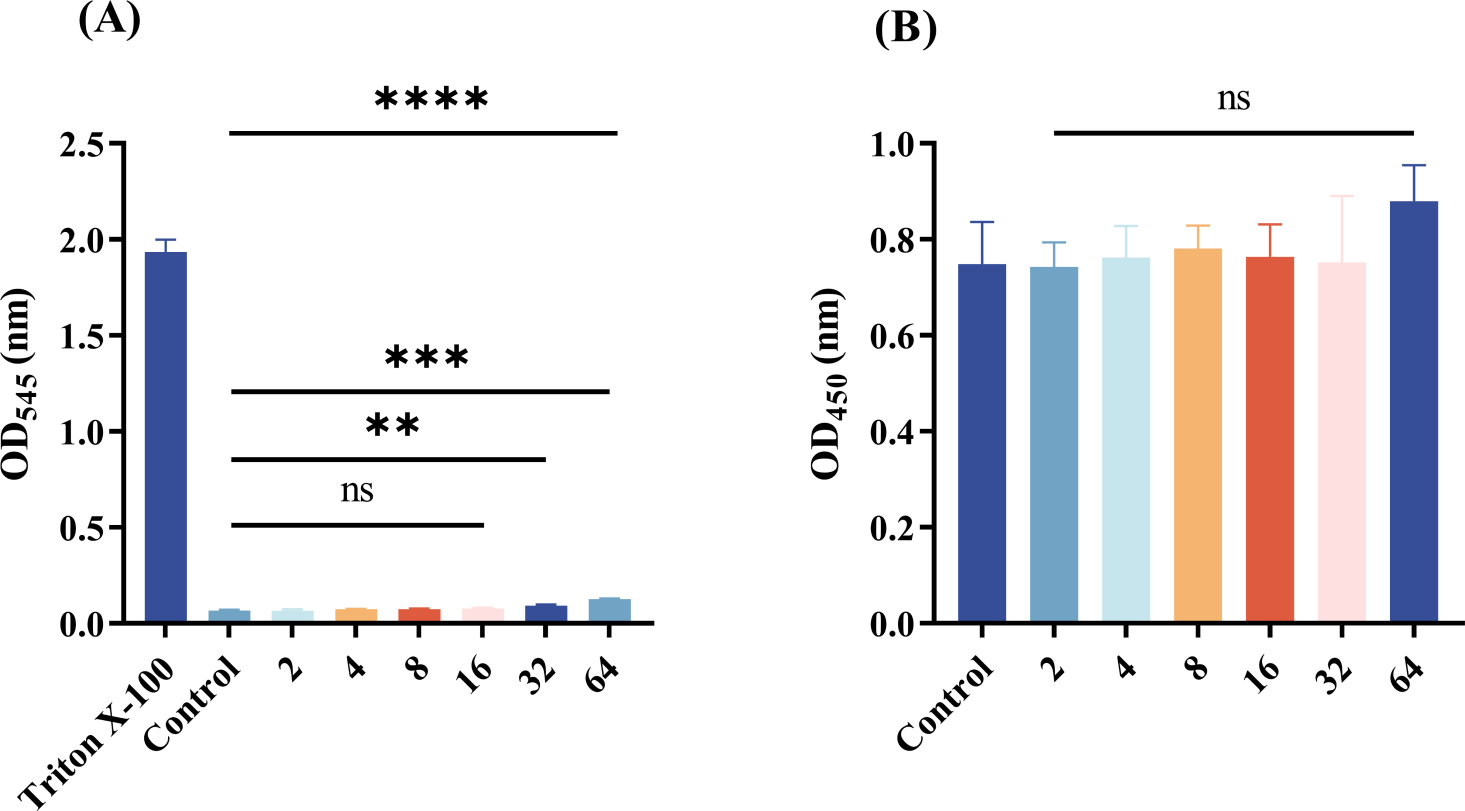
(**A**) SMAP29 hemolysis as a function of concentration (1–64 μg/mL). Positive hemolysis is 5% or greater. (**B**) SMAP29 concentration-induced cytotoxicity on RAW 264.7 cells. *P* < 0.01 (**), *P* < 0.001 (***), and *P* < 0.0001 (****); ns, not significant.

As seen in Fig. 6A, erythrocytes were also utilized to assess SMAP29 hemolysis. Considering the control group, at doses up to 32 μg/mL, SMAP29 only exhibited modest hemolytic activity (*P* < 0.05) and almost no hemolytic activity when it was less than or equal to 16 μg/mL. The MIC value of SMAP29 to COL-R GNB is much lower than this result, which indicates that SMAP29 has certain application potential *in vivo*. Figure 6B illustrates the cytotoxicity results of SMAP29 screening on RAW 264.7 cells to assess its safety. At concentrations as high as 64 μg/mL, SMAP29 was approximately identical in quantity to the control group.

### Effect of SMAP29 on pro-inflammatory cytokine and *in vivo* antibacterial effect evaluation

**Fig 7:**
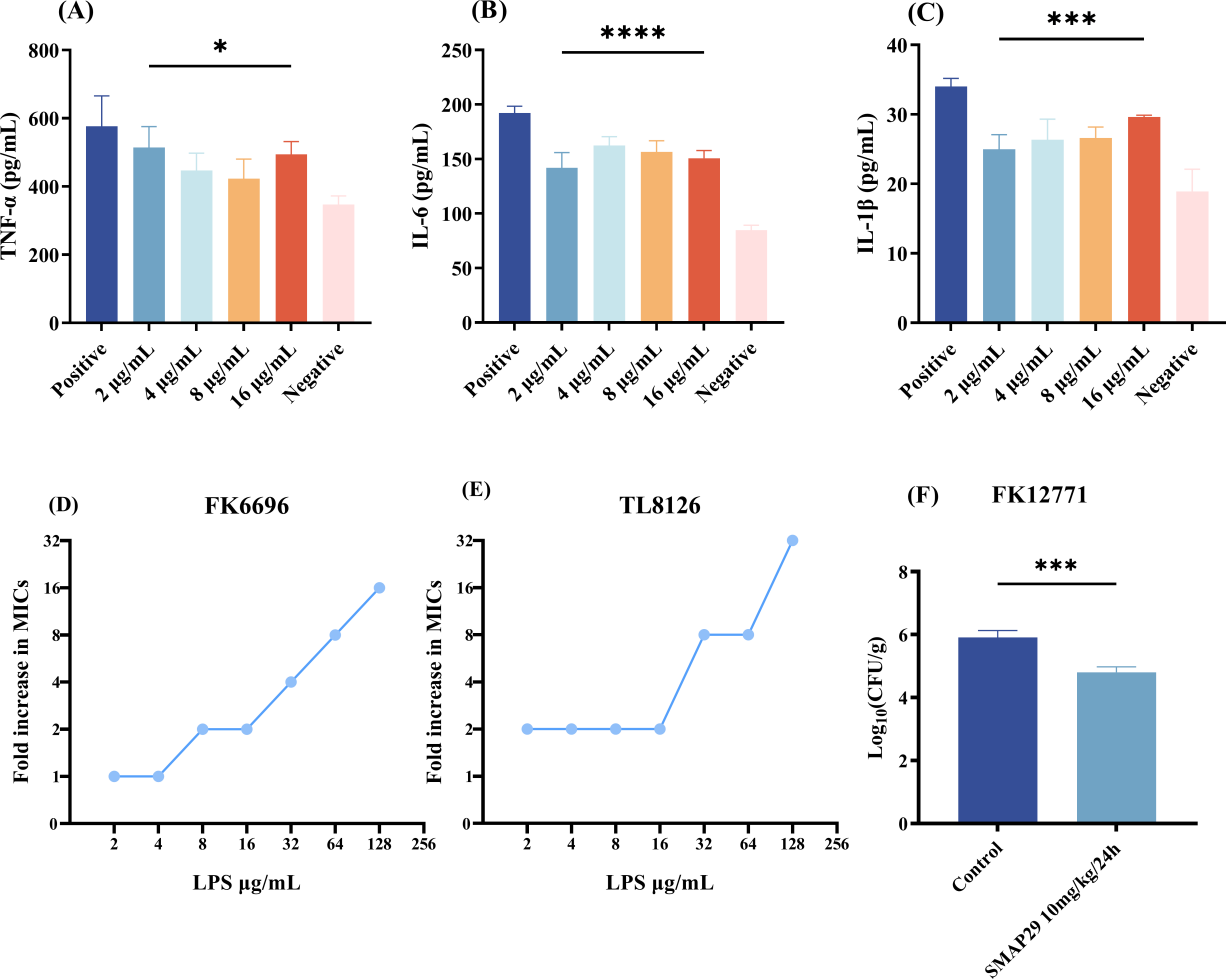
In *vitro* immunomodulation, LPS antagonism, and in *vivo* efficacy of SMAP29. (**A–C**) SMAP29 attenuates LPS-induced release of pro-inflammatory cytokines (TNF-α, IL-1β, and IL-6) from RAW 264.7 macrophages, as measured by ELISA. (**D and E**) Neutralizing effect of exogenous LPS on SMAP29 activity. The fold change in MIC of SMAP29 against *Klebsiella pneumoniae* FK6696 and *Pseudomonas aeruginosa* TL8126 is shown upon the addition of LPS. (**F**) In *vivo* antibacterial efficacy. Bacterial burden in thigh muscles of neutropenic mice infected with *Klebsiella pneumoniae* FK12771, measured 24 h post-treatment with SMAP29. Data are presented as mean ± SD from two independent experiments, with a total of eight mice per group (*n* = 8). Each experiment included four biological replicates per group. *P* < 0.05 (*), *P* < 0.001 (***), and *P* < 0.0001 (****).

Endotoxins, which are produced by infections caused by bacteria, drive macrophages to release a great deal of pro-inflammatory cytokines. Figure 7A through C demonstrates that SMAP29 considerably reduced IL-6, TNF-α, and IL-1β levels at 2–16 µg/mL versus positive controls (LPS alone). LPS may be the target of SMAP29 and participate in its anti-biofilm activity and anti-inflammatory effect. Competitive tests employing various amounts of exogenous LPS were carried out to further validate SMAP29’s binding capacity to LPS. The concentration-dependent rise in the MIC values of the two bacterial strains demonstrated that exogenous LPS weakened contacts between SMAP29 and the bacterial cell membrane by competing with SMAP29 (Fig. 7D and E).

To validate the in *vivo* antibacterial efficacy, we established a mouse thigh infection model. And to evaluate the therapeutic efficacy of SMAP29 during the acute phase of infection, mice were euthanized, and bacterial burdens were assessed at a single, critical time point post-infection (24 h). At 24 h post-infection, treatment with SMAP29 reduced the bacterial burden in thigh muscle by 0.7–1.5 log₁₀ CFU/g compared to the untreated control, in which bacterial proliferation proceeded unimpeded (*P <* 0.05, using strain FK12771; Fig. 7F).

## DISCUSSION

New, potent, broad-spectrum antimicrobial remedies for COL-R GNB infection are urgently needed (20, 21). In the process of exploring new antibacterial strategies, AMPs are considered a powerful weapon against MDR bacteria owing to poor bacterial resistance and superior antibacterial action (22, 23). However, the healthcare application of AMPs has intrinsic drawbacks, like cytotoxicity, minimal antibacterial action, and pricey production costs (9, 24).

The primary mechanism of SMAP-29 involves high-affinity, electrostatic interaction with LPS on the bacterial outer membrane (25), leading to rapid disruption and bactericidal activity within 30 min. This initial targeting is selective; bacterial membranes possess a higher negative charge density due to LPS and phospholipids, whereas mammalian cell membranes are more neutral (26). Consequently, while earlier studies reported cytotoxicity at high concentrations (>6 to 25 μM) (27), the variant in our study operates at substantially lower, effective antibacterial concentrations (MIC: 0.077–2.46 μM), demonstrating a wide therapeutic window. This underscores that cytotoxicity is a concentration-dependent, off-target effect that can be dissociated from its primary LPS-targeted mechanism.

SMAP29 shares a membrane-targeting premise with colistin but differs structurally (amphipathic alpha-helix vs. cyclic peptide with a fatty acid chain) and functionally (28). A critical functional distinction is SMAP-29’s ability to suppress pro-inflammatory cytokine release (e.g., IL-6, TNF-α). This immunomodulatory effect is likely due to its high-affinity neutralization of free LPS, thereby preventing LPS-mediated immune hyperactivation, rather than direct elimination of bacteria (25). This dual action—direct killing and mitigation of harmful inflammation—represents a potential therapeutic advantage.

We acknowledge that the membrane permeability increases observed in our NPN/PI assays (approximately 1.3-fold to 1.6-fold at 2× MIC) are modest compared to the positive control. However, complete membrane solubilization is not required for bacterial killing; cationic AMPs like SMAP-29 can act through transient pore formation or proton motive force disruption—events that do not necessitate extensive membrane damage (29, 30). The concentration-dependent and statistically significant increases at and above the MIC are consistent with such a mechanism. These subtle perturbations may allow peptide access to intracellular targets (31), leading to subsequent ROS generation (up to 1.6-fold) and rapid bacterial killing.

Similarly, the reductions in pro-inflammatory cytokines (TNF-α, IL-1β, and IL-6) were modest but significant. We interpret this as a physiologically relevant immunomodulatory effect. In a therapeutic context, complete cytokine ablation could impair host defense, whereas partial dampening of hyper-inflammation, combined with direct bacterial killing, may represent an optimal balance—reducing sepsis-associated pathology while preserving essential immune functions.

A biphasic response—rapid killing followed by bacterial regrowth at later time points—was observed. This is not uncommon for membrane-targeting peptides and may result from peptide degradation, phenotypic tolerance in a bacterial subpopulation, or adaptive stress responses (32). Crucially, this regrowth highlights the challenge of complete eradication with monotherapy but does not negate the potent initial efficacy. Future strategies like combination therapy or peptide engineering to enhance stability could address this limitation (33).

While the time-kill kinetics data (Fig. S1) clearly establish the rapid bactericidal activity of SMAP29—achieving a >3 log₁₀ CFU/mL reduction within 10 min—we acknowledge that these endpoint measurements do not fully capture the real-time sequence of events leading to bacterial death. Real-time monitoring of membrane permeability (e.g., propidium iodide uptake) and membrane potential (e.g., using DiSC₃-5) during the first 0–10 min of peptide exposure would provide critical mechanistic insight. This important question remains unresolved in the current study and represents a key direction for future investigation. Employing flow cytometry or live-cell fluorescence microscopy to simultaneously track membrane integrity and viability at the single-cell level would be particularly informative, allowing discrimination between all-or-none killing events and gradual membrane destabilization.

The translational potential of SMAP-29 was evidenced in a neutropenic mouse thigh infection model, where a tolerated dose significantly reduced the bacterial burden of MDR *K. pneumoniae*. However, we acknowledge two key limitations of this in *vivo* study: (i) assessment at a single time point, which precludes a dynamic pharmacokinetic/pharmacodynamic profile and (ii) the absence of a standard-of-care antibiotic (e.g., Imipenem) as a positive control for direct efficacy comparison. The primary goal here was to establish proof-of-concept for SMAP-29’s *in vivo* activity against COL-R infections, setting the stage for future comparative and time-course studies.

Importantly, our study revealed that the antimicrobial activity of SMAP-29 against clinically isolated COL-R strains exhibited heterogeneity, rather than universal failure. This indicates that bacterial resistance to colistin does not automatically confer cross-resistance to SMAP-29. We hypothesize that this heterogeneity is linked to the diversity of LPS modifications underlying COL-R. Resistance to colistin primarily arises from the addition of positively charged groups (e.g., phosphoethanolamine or 4-amino-4-deoxy-L-arabinose) to lipid A, which repel the cationic drug, or, more rarely, from complete LPS loss (34). Although SMAP-29 is also cationic, its distinct mechanism of action (e.g., greater hydrophobicity, ability to induce membrane thinning) may allow it to circumvent the effects of certain LPS modifications that are effective against colistin (26). Therefore, specific alterations may preferentially interfere with colistin binding while remaining vulnerable to SMAP-29 penetration. Conversely, strains resistant to both agents may have undergone more fundamental membrane alterations, conferring broad-spectrum resistance to membrane-active peptides.

It must be noted that this study did not perform systematic genotypic screening for LPS modification genes or detailed chemical structural analysis of lipid A in the COL-R strains. Therefore, we cannot precisely correlate the SMAP29-sensitive phenotype of a specific strain with a particular LPS mutation. However, the phenotypic heterogeneity observed herein is of significant indicative value: it suggests that SMAP29’s mechanism of action differs from that of colistin and reveals its potential to overcome specific types of colistin resistance. Future research should employ lipid A mass spectrometry analysis and genetic complementation experiments to directly identify the key LPS chemical modifications associated with SMAP29 susceptibility, thereby elucidating the molecular basis of this selective activity.

In conclusion, SMAP-29 presents a promising lead candidate against COL-R GNB, combining rapid membrane-targeting killing with anti-inflammatory potential and a demonstrable therapeutic window. The future of its development lies in rational peptide engineering to further minimize any off-target effects and enhance metabolic stability. Our findings strongly support continued investigation of SMAP-29 and its analogs as a potential new class of antibiotics to combat multidrug-resistant infections.

### Conclusion

Our study demonstrates that the natural antimicrobial peptide SMAP-29 exerts potent activity against colistin-resistant gram-negative bacteria through a multi-faceted mechanism. It primarily acts by targeting and disrupting the bacterial outer membrane via high-affinity interaction with LPS, leading to rapid bactericidal effects and significant impairment of biofilm formation. Beyond direct killing, SMAP-29 also exhibits immunomodulatory potential by neutralizing free LPS, thereby attenuating the subsequent pro-inflammatory cytokine response in macrophages. Critically, these in *vitro* findings were corroborated by in *vivo* efficacy in an infection model. Collectively, the evidence positions SMAP-29 as a promising lead candidate worthy of further development in the fight against multidrug-resistant bacterial infections.

## MATERIALS AND METHODS

### Antibacterial peptide and antibacterial drugs

Chinese company Nanjing Yuanpeptide Biotechnology Co., Ltd. synthesized SMAP29. The amino acid sequence of SMAP29 is RGLRRLGRKIAHGVKKYGPTVLRIIRIAG (molecular weight: 3,286) (17). Amikacin, ceftazidime, cefepime, imipenem, ciprofloxacin, levofloxacin, gentamicin, tobramycin, and chloramphenicol are among the antibiotics used in this investigation. They were acquired from Kang Tai Biotechnology Co., Ltd. in Wenzhou, Zhejiang, China. Mueller-Hinton broth (MHB), manufactured by Thermo Fisher Scientific in the United States, was used for antimicrobial susceptibility testing.

### Bacterial isolates and growth conditions

This investigation identified 32 gram-negative clinical strains from the First Affiliated Hospital of Wenzhou Medical University. MALDI-TOF-MS (BioMerieux, France) identified the isolates. Isolates were frozen at −80°C in LB broth with 30% glycerol after collection. For controls, the National Center for Clinical Laboratories provided *E. coli* ATCC 25922, *P. aeruginosa* ATCC 27853, *K. pneumoniae* ATCC 700603, and *A. baumannii* ATCC 19606.

### Antimicrobial susceptibility testing

The MICs of SMAP29 and common antibiotics were measured against 32 clinical GNB isolates in Mueller-Hinton broth (MHB). The MIC was determined via broth microdilution in 96-well polystyrene plates (brand is BKMAM, Changde Bikeman Biotechnology Co., Ltd., China) following CLSI guidelines (M100, 34th ed.). SMAP29 and antibiotics were serially diluted in Mueller-Hinton Broth (MHB) across a concentration range of 0.01–256 µg/mL. Overnight bacterial cultures were standardized to a 0.5 McFarland standard and diluted 1:100 in MHB. Subsequently, the bacterial suspension of 100 µL was added to 100 µL of each drug dilution. The plates were incubated at 37°C for 16–18 h. The MIC was recorded as the lowest concentration that completely inhibited visible growth. To validate our findings against potential plate adsorption artifacts, parallel MIC assays were performed using polypropylene microtiter plates (brand is LABSELECT, Beijing Lableteck Technology Co., Ltd., China) for representative strains, as recommended by Wiegand et al. (35). The results, shown in Table S1, confirmed the values obtained with standard polystyrene plates.

All experiments were performed in three independent biological replicates.

### Time kill test

The time-kill assay was conducted according to the published method, with minor changes (36, 37). A time-kill assay was performed to assess the bactericidal kinetics of SMAP29. Bacterial suspensions (1 × 10⁶ CFU/mL in Mueller-Hinton Broth) were treated with SMAP29 at 1/4×, 1/2×, 1×, and 2× MIC concentrations, with phosphate-buffered saline (PBS) as a negative control. Cultures were incubated at 37°C with shaking (200 rpm). At designated time points (0 and 10 min) and (0, 0.5, 1, 2, 4, 6, 8, 12, and 24 h), aliquots were serially diluted in saline and plated onto LB agar for viable colony counting after 16–18 h of static incubation at 37°C. Each condition was tested in quadruplicate. The lower limit of detection was 100 CFU/mL (2 log_10_ CFU/mL).

### Biofilm inhibition and destruction test

The biofilm inhibition experiment was carried out as previously reported (38). Biofilm formation was quantified using a crystal violet (CV) assay. Eight polymyxin-resistant gram-negative strains were treated with SMAP29 at sub-inhibitory to inhibitory concentrations (1/4×, 1/2×, 1×, and 2× MIC) in 96-well plates. Following a 24-h incubation for *Klebsiella pneumoniae* and *Pseudomonas aeruginosa*, or a 48-h incubation for *Escherichia coli* and *Acinetobacter baumannii*, non-adherent cells were removed by washing twice with PBS. The adherent biofilms were stained with 1% CV for 15 min, washed, and the bound dye was solubilized with anhydrous ethanol. Biofilm biomass was quantified by measuring the absorbance at 595 nm using a microplate reader (Multiskan FC, Thermo Fisher Scientific). All experiments were performed with three independent biological replicates.

Both biofilm development inhibition and elimination used the same strains. A 200 μL bacterial solution aliquot was injected into 96-well plates. Planktonic cells were removed by washing the wells with PBS after 24–48 h. With PBS to eliminate plankton. After that, 200 μL of drug solutions and 200 μL of drug-free broth were added as controls and incubated for 24 h. The biofilm destruction and inhibition tests used the same CV staining, elution, and absorbance measurements.

### Scanning electron microscope

We used silicon wafers (3 × 3 mm) in 24-well plates to construct biofilm-forming surfaces for SEM experiments (39). After adding 10 μL of fresh cell suspension to 990 μL of MHB with SMAP29, the mixture was incubated at 37°C for 24 h. After incubation, the wafers were gently washed three times with PBS, fixed with 2.5% glutaraldehyde, and dehydrated with ethanol gradients (30%, 50%, 70%, and 100%) for 5 min at each concentration. After air drying and gold sputtering, the fully dehydrated samples were examined by SEM (Hitachi SU8010, Japan).

### Membrane permeability analysis and active oxygen detection

Four distinct strains were randomly selected from the tested COL-R GNB (FK6696, DC19144, TL8126, and BM7994) employed for ROS detection and membrane permeability tests, according to the previously reported protocol to detect reactive oxygen species (40). Bacterial membrane permeability was assessed using fluorescent probes specific for the outer and inner membranes. Untreated bacteria and bacteria treated with 0.1% Triton X-100 served as negative and positive controls, respectively. Bacterial membrane permeability was assessed using fluorescent probes specific for the outer and inner membranes (41, 42). For outer membrane integrity, bacterial suspensions (OD_₆₀₀_ = 0.3–0.4) treated with SMAP29 (1/4×, 1/2×, 1×, and 2× MIC) were incubated with NPN (30 µg/mL) for 30 min. For inner membrane integrity, bacteria treated under the same conditions were incubated with PI (50 µg/mL) for 1 h. Following incubation with either probe, cells were harvested by centrifugation (4,000 × *g*, 5 min) to remove unbound dye, resuspended in buffer, and transferred to a 96-well plate (200 µL per well, *n* = 3). Fluorescence was measured immediately for NPN (ex/em: 350/420 nm) and after the PI incubation period (ex/em: 535/615 nm) using a microplate reader.

Intracellular ROS were detected using the cell-permeable fluorescent probe 2′,7′-dichlorodihydrofluorescein diacetate (DCFH-DA) (43). Untreated bacteria served as the negative control. Bacterial cultures were loaded with 10 µM DCFH-DA at 37°C for 30 min in the dark, followed by three washes with PBS to remove extracellular probe. The labeled bacteria were then treated with SMAP29 (1/4×, 1/2×, 1×, and 2× MIC) for 30 min at 37°C. Cells were harvested by centrifugation (4,000 × *g*, 5 min), resuspended in PBS, and transferred to a 96-well plate (200 µL per well, *n* = 3). Fluorescence intensity (excitation/emission: 488/525 nm) was measured using a multifunctional microplate reader.

### CLSM live/dead staining

PI (red, Solarbio, Beijing, Shanghai) and SYTO 9 (green, Thermo Fisher Scientific, USA) were used for fluorescent dual labeling of the bacteria in the biofilm (44). DC19144 (1.5 × 10^6^ CFU/mL) was cultivated in MHB broth with medicines at a concentration of 1/2× MIC using confocal dishes as containers, and it was incubated for 24 h at 37°C. Following the recommended protocol, live/dead staining was carried out after planktonic bacteria were eliminated. After washing the biofilm with sterile PBS twice to remove excess dye, the biofilm was imaged in two dimensions by confocal laser microscope (Leica, Japan).

### *In vivo* evaluation of synergy in a mouse infection model

To test SMAP29’s *in vivo* effectiveness, a modified neutropenic mouse thigh infection model was used (45). Experimental strain FK12771 was chosen. Two independent experiments were performed, with a total of eight mice per group (*n* = 8). Animals received 150 mg/kg intraperitoneal cyclophosphamide for 3 days to develop neutropenic animals. Each mouse received a 100 μL bacterial solution (1.5 × 10^7^ CFU/mL) injection into the back thigh muscle. After 2 h of bacterial inoculation, the experimental groups received 5 mg/kg, 10 mg/kg, and 20 mg/kg SMAP29 intraperitoneally. After 24 h, mice were euthanized, and thigh muscles were homogenized. Bacterial colony counts were done by plating serial dilutions of homogenates on MHB and incubating at 37°C for 24 h.

### Impact of external lipopolysaccharide on the function of SMAP29

The antagonistic effect of exogenous LPS on SMAP29 was evaluated using a checkerboard titration assay (46). LPS was sourced from Solabo (Meigu Bio-Tech Co., Ltd.). Bacterial suspensions (Klebsiella pneumoniae FK6696 and Acinetobacter baumannii TL8126; 1 × 10^6^ CFU/mL) were co-incubated with twofold serial dilutions of LPS (0–256 µg/mL) and SMAP29 (0–32 µg/mL) in 96-well plates at 37°C for 18–20 h. The MIC of SMAP29 across the LPS concentration gradient was then determined. All experiments were performed with three independent biological replicates.

### Hemolytic activity and cytotoxicity assay

We used erythrocytes to assess erythrocyte hemolysis (47). Fresh human blood was collected and centrifuged to remove plasma. Erythrocytes were washed three times with PBS and then diluted with PBS to a 10% (vol/vol) stock suspension. For the assay, 300 µL of the 10% erythrocyte suspension was mixed with 300 µL of PBS containing various concentrations of SMAP29 (2–64 μg/mL) in microcentrifuge tubes, yielding a final erythrocyte concentration of 5% (vol/vol). The tubes were incubated in a CO₂ incubator at 37°C for 2 h with gentle agitation. After incubation, samples were photographed to visually document hemolysis. The tubes were then centrifuged at 3,000 rpm for 5 min, and 200 µL of the supernatant from each tube was carefully transferred to a 96-well plate in triplicate. Hemolysis was quantified by measuring the absorbance of the released hemoglobin at 545 nm using a Multiskan FC microplate reader.

Hemolysis rate (%) = (OD_540_ test − OD_540_ negative)/(OD_540_ positive − OD_540_ negative).

The safety of SMAP29 was tested with RAW 264.7 cells using a previously established approach (39). Cells were grown at 37°C for 24 h with 5% CO2 after adding 10 µL of SMAP29 to obtain a final concentration of 2–64 µg/mL. CCK-8 of 10 µL was added and incubated in the dark for 1 h. Finally, a Multiskan FC microplate reader evaluated each well’s 450 nm absorbance.

### The cytokine concentration was measured by ELISA

Adhering to the established protocol, RAW 264.7 cells were stimulated with 1 µg/mL LPS (48). Cells were supplied with SMAP29 therapy for 4 h at various doses. Then the supernatant was taken. TNF-α, IL-6, and IL-1β levels were measured using an ELISA kit (J&L Biological, Inc.) as per the manufacturer’s instructions.

### Statistical analysis

The findings of all triplicate trials were examined using Prism 8 (GraphPad Software Inc., CA, USA). The data were shown as mean ± SD. Statistical significance was established using two-sample *t* tests (*P* < 0.05 (*), *P* < 0.01 (**), *P* < 0.001 (***), and *P* < 0.0001 (****).

## Data Availability

The authors confirm that the data supporting the findings of this study are available within the article and its supporting information materials.

## SUPPLEMENTAL MATERIAL

Figure S1 spectrum.02808-25-s0001.docx Early time-kill kinetics of SMAP29 against representative Gram-negative bacteria.

Graphical abstract spectrum.02808-25-s0002.tif Visual depiction of the study outline.

Table S1 spectrum.02808-25-s0003.docx MICs of SMAP29 against 32 COL-R clinical isolates in polystyrene vs. polypropylene plates.

ASM does not own the copyrights to Supplemental Material that may be linked to, or accessed through, an article. The authors have granted ASM a non-exclusive, world-wide license to publish the Supplemental Material files. Please contact the corresponding author directly for reuse.
