# Supplementary material for: SMAP29: an antibacterial peptide that possesses anti-inflammatory and fast bactericidal actions against colistin-resistant gram-negative bacteria
Source: Microbiol Spectr. 2026 May 5;14(6):e02808-25. doi: 10.1128/spectrum.02808-25 (PMC13228009; doi:10.1128/spectrum.02808-25)
Supplement: Figure S1 — Early time-kill kinetics of SMAP29 against representative Gram-negative bacteria. [file spectrum.02808-25-s0001.docx]

****
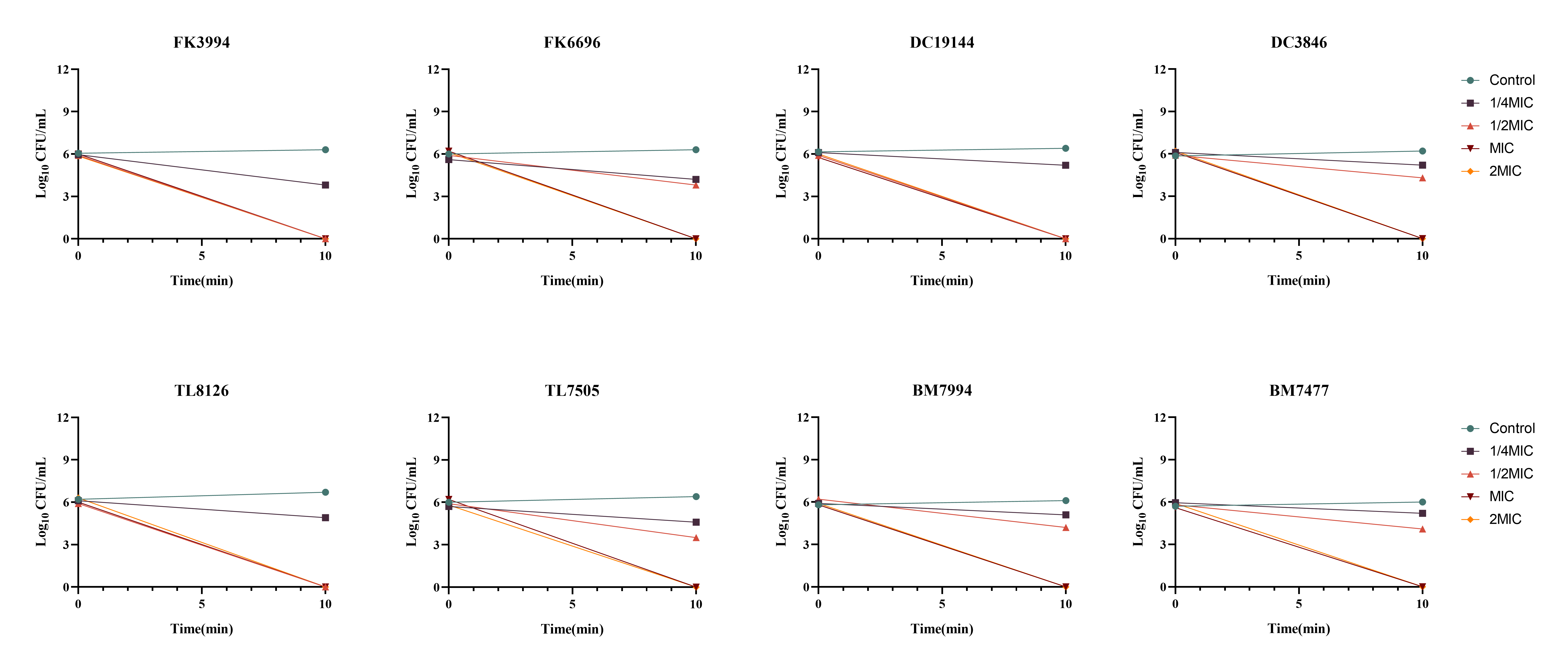
**Figure S1** Early Time-kill kinetics of SMAP29 against Gram-negative bacteria (n=3). SMAP29 was tested against *Klebsiella pneumoniae* (FK), *Escherichia coli*(DC), *Pseudomonas aeruginosa* (TL), and *Acinetobacter baumannii*(BM) at concentrations of 1/4 ×, 1/2 ×, 1 ×, and 2 × MIC.
