# Supplementary figures and images for: SMAP29: an antibacterial peptide that possesses anti-inflammatory and fast bactericidal actions against colistin-resistant gram-negative bacteria
Source: Microbiol Spectr. 2026 May 5;14(6):e02808-25. doi: 10.1128/spectrum.02808-25 (PMC13228009; doi:10.1128/spectrum.02808-25)

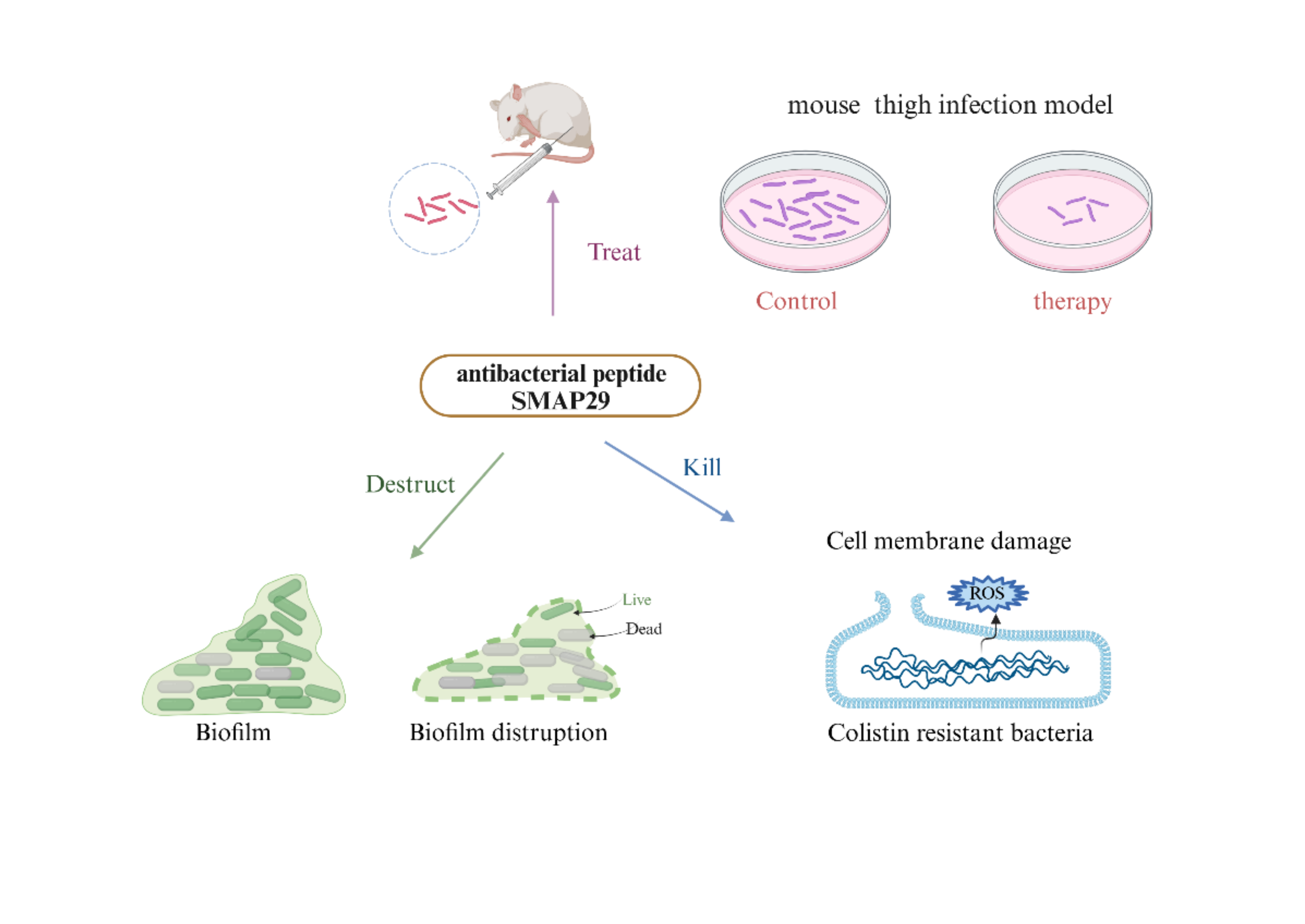

Supplement: Graphical abstract — Visual depiction of the study outline. [file spectrum.02808-25-s0002.tif]
