# Supplementary material for: SMAP29: an antibacterial peptide that possesses anti-inflammatory and fast bactericidal actions against colistin-resistant gram-negative bacteria
Source: Microbiol Spectr. 2026 May 5;14(6):e02808-25. doi: 10.1128/spectrum.02808-25 (PMC13228009; doi:10.1128/spectrum.02808-25)
Supplement: Table S1 — MICs of SMAP29 against 32 COL-R clinical isolates in polystyrene vs. polypropylene plates. [file spectrum.02808-25-s0003.docx]

****Table S1 MICs of SMAP29 against 32 COL-R clinical isolates in polystyrene vs polypropylene plates****

| **Strain** | **MIC in Polystyrene (µg/mL)** | **MIC in Polypropylene (µg/mL)** |
| --- | --- | --- |
| **^DC90^** | ^2^ | ^2^ |
| **^DC19840^** | ^0.5^ | ^0.5^ |
| **^DC18824^** | ^0.25^ | ^0.25^ |
| **^DC3846^** | ^0.5^ | ^0.5^ |
| **^DC5286^** | ^0.25^ | ^0.25^ |
| **^DC19144^** | ^2^ | ^1^ |
| **^DC19526^** | ^2^ | ^1^ |
| **^DC19829^** | ^1^ | ^0.5^ |
| **^FK1913^** | ^2^ | ^1^ |
| **^FK3994^** | ^2^ | ^2^ |
| **^FK6556^** | ^0.5^ | ^0.25^ |
| **^FK6663^** | ^1^ | ^0.5^ |
| **^FK6696^** | ^4^ | ^2^ |
| **^FK11237^** | ^1^ | ^0.5^ |
| **^FK12771^** | ^2^ | ^1^ |
| **^FK12716^** | ^0.5^ | ^0.5^ |
| **^BM1342^** | ^0.5^ | ^0.5^ |
| **^BM1412^** | ^1^ | ^0.5^ |
| **^BM2431^** | ^1^ | ^1^ |
| **^BM7477^** | ^0.5^ | ^0.5^ |
| **^BM2349^** | ^1^ | ^0.5^ |
| **^BM7994^** | ^1^ | ^1^ |
| **^BM2370^** | ^1^ | ^1^ |
| **^BM8014^** | ^0.5^ | ^0.5^ |
| **^TL2314^** | ^1^ | ^1^ |
| **^TL7333^** | ^4^ | ^2^ |
| **^TL7440^** | ^2^ | ^2^ |
| **^TL7505^** | ^4^ | ^2^ |
| **^TL7548^** | ^1^ | ^0.5^ |
| **^TL2917^** | ^1^ | ^1^ |
| **^TL7929^** | ^4^ | ^2^ |
| **^TL8126^** | ^4^ | ^2^ |
